# Early initiation of sexual activity: a risk factor for sexually transmitted diseases, HIV infection, and unwanted pregnancy among university students in China

**DOI:** 10.1186/1471-2458-9-111

**Published:** 2009-04-22

**Authors:** Qiaoqin Ma, Masako Ono-Kihara, Liming Cong, Guozhang Xu, Xiaohong Pan, Saman Zamani, Shahrzad Mortazavi Ravari, Dandan Zhang, Takayuki Homma, Masahiro Kihara

**Affiliations:** 1 Center for Disease Control and Prevention of Zhejiang Province, Hangzhou, 310051, PR China; 2 Department of Global Health and Socio-epidemiology, Kyoto University School of Public Health, Kyoto, 606-8501, Japan; 3 Center for Disease Control and Prevention of Ningbo Municipality, Ningbo, 315010, PR China; 4 Kanazawa University Graduate School of Natural Science & Technology, Kanazawa city, Ishikawa, 920-1192, Japan

## Abstract

**Background:**

To explore any association between the timing of the initiation of sexual activity and sexual behaviors and risks among university students in China.

**Methods:**

Data were derived from a cross-sectional study on sexual behavior among university students conducted in Ningbo municipality, China, at the end of 2003. Students completed a self-administered, structured questionnaire. Of 1981 sexually active male students, 1908 (96.3%) completed the item for timing of the initiation of sexual activity and were included in bivariate trend analyses and multiple logistic regression analyses to compare the association between this timing and sexual behavior and risks.

**Results:**

Male early sexual initiators had a significantly higher risk profile, including a significantly higher proportion reporting non-regular partners (i.e., casual or commercial partners), multiple partners, diagnosis with a sexually transmitted disease (STD), partner history of pregnancy, partner history of induced abortion, and less condom and oral contraceptive use, compared with late initiators. Multivariate analyses confirmed the increased likelihood of these risks in early initiators versus late initiators, other than partner type during the last year.

**Conclusion:**

Our results showed that, compared to late initiators, people who initiated sexual activity early engaged in more risky behaviors that could lead to elevated risks of unwanted pregnancies and STDs or human immunodeficiency virus infection. Sex-education strategies should be focused on an earlier age, should include advice on delaying the age of first sexual activity, and should target young people who continue to take sexual risks.

## Background

Sexual activity rates in Chinese university students are still low; studies in different regions have shown that the range of those engaging in sexual activity is between 5 and 20% [1-5]. However, with the great changes in the economy and culture in China since the start of its open-door policies in the 1970s and the economic reforms of the 1980s, the sexual behaviors and attitudes of Chinese people are changing rapidly, becoming more active and liberal [6-8]. More and more young people are having sex at an earlier age, and they generally do not protect themselves [9,10]; indeed, the age of sexual activity onset in university students has decreased [4]. At the same time, sexually transmitted diseases (STDs) and a human immunodeficiency virus (HIV) epidemic have spread rapidly in China in recent years [11]. National reports in 2005 and 2007 indicated that HIV/acquired immunodeficiency syndrome (AIDS) is still on the rise, spreading from high-risk groups to the general population, and the proportion of sexual transmission among HIV-infected persons is increasing each year [12,13]. STD incidence is one of the highest among all notifiable infectious diseases [14]. From 1987 to 2006, the reported incidence of syphilis increased from .08 to 13.35 per 100,000 people [13]. Previous reports have indicated that, although the rate of sexual activity in Chinese university students is generally low, some sexually active students engage in much risky behaviors, including very low condom use, very low contraceptive use generally, and sex with multiple and commercial partners [2-5]. Such behaviors make them vulnerable to STD/HIV infection and pregnancy. With more people being likely to initiate sexual activity earlier than ever before, often becoming sexually active in the adolescent period, there is serious concern regarding the health consequences of such early sexual initiation. However, information about the behavioral characteristics of those who initiate sexual activity early and the risks among young Chinese people, including university/college students, is very limited. If safe-sex education programs are to be successful in the future for Chinese students, a better understanding of early onset sexual activity and subsequent sex-related risks and behaviors among young people is necessary.

The purpose of this study was to explore the relationship between early initiation of sexual activity and risks to sexual and reproductive health among a group of sexually active male university students in an eastern Chinese city, and to understand how best to tailor effective sex education for this sort of population.

## Methods

### Setting and Procedures

This research was conducted in Ningbo municipality, a large coastal city in Zhejiang Province in eastern China that is home to two universities, both of which participated in the study. The research methods have been introduced elsewhere [8]. Briefly, an instrument was developed based on a review of domestic and international literature, modified by qualitative studies that included 11 in-depth interviews and four focus groups among students from the two universities. The revised instrument was pilot tested in a group of 50 students at one of the universities. Then, the instrument's reliability was evaluated in 89 of 160 college students recruited from another city, who could be matched between the two tests with a one-week interval. The survey was conducted in November and December, 2003. All grade I–IV students at the two universities were requested by university staff and student leaders to come to classrooms at specified times outside normal lecture hours to fill out a self-administered and anonymous questionnaire. The trained staff of the local Centers for Disease Control (CDC) and the two universities collected data in the field.

### Participants

Of the total of 29,409 eligible participants at the two universities, 22,940 (78.0%) actually responded; 447 were eliminated from the analysis due to evident invalid responses. Thus, 22,493 students responded validly (valid response rate of 76.5%). Of the 22,493 respondents, 1981 (17.6%) men and 963 (8.6%) women reported being sexually active. In this paper, sexually active male students who responded to the question, "in which school period did you initiate sexual activity," were included in the analysis, resulting in a final sample size of 1908 men, 96.3% of all sexually active male students. Female students were excluded because only a small number of them were sexually active before high school (i.e., in primary school or junior high school). We categorized sexually active male students into three groups according to the timing of their reported sexual initiation: those who initiated sex before high school (BHS initiator), those who initiated sex in high school (HS initiator), and those who reported initiating sex at university (Univ initiator).

### Ethical assessment

The research protocol, including the questionnaire, was ratified by the institutional review processes of the local education board, the two universities involved, and Zhejiang Provincial Center for Disease Prevention and Control. Participants were informed of the purpose and the methods of the study. All were welcome to participate, with no advantage or disadvantage for participation or non-participation. These policies were also printed on the front page of the questionnaire.

### Statistical analyses

Epi-Info (Version 6.0, CDC, Atlanta, GA) and SPSS for Windows (Version 12.01; SPSS Inc., Chicago, IL) were employed for the analyses. Differences in the prevalence of sexual behaviors and their consequences and their relationship to the timing of sexual activity onset were assessed using a chi-square test for linear trends in proportion. Those variables exhibiting a significant linear trend, where greater risk was associated with the timing of sexual activity onset, were further assessed using multiple logistic regression analysis, adjusted for possible confounding by university, grade, faculty, hometown area, and reported family economic status, with sexual activity initiation in university as a reference. Lifetime sexual behavior and that during the the most recent year were further adjusted for the duration of sexually active life, calculated by subtracting the age of first sexual activity from the current age, to adjust for confounding by different lengths of sexually active life versus the timing of sexual initiation. Adjusted odds ratios and 95% confidence intervals summarizing any association between the selected variables and the timing of sexual initiation were calculated for each category. A *P *value of less than 0.05 was deemed to indicate statistical significance.

## Results

### Socio-demographics

The percentages of the 1908 sexually active male students designated as BHS, HS, and Univ initiators were 6.0% (115), 36.9% (705), and 57.0% (1088), respectively (Table 1). The age range of all participants was 17–25 (median, 21). About three-quarters of the sexually active males among the BHS and HS initiators were aged over 19 years old; this age group made up 93.3% of Univ initiators. Of the students, 94.8% of BHS and 95.7% of HS initiators were in Grades I–III, whereas 91.5% of Univ initiators were in Grades II–IV (Table 1). For all timings of sexual initiation, the majority of sexually active males perceived their family economic status to be mid-level, and the majority came from a town or city. The age range for initiating sexual activity in all participants was 10–24 (median, 20). The mean ages at first sexual activity for BHS, HS, and Univ initiators were 15.53 (SD, 1.94), 18.38 (SD, 1.25), and 20.44 (SD, 1.16), respectively.

**Table 1:** Socio-demographic characteristics of sexually active males by timing of sex initiation.

Variables	Timing of sex initiation		
	
	BHS (n = 115)^a^	HS (n = 705)^a^	Univ (n = 1088)^a^
Current age			
≤ 19	21.4	24.3	6.7
> 19	78.6	75.7	93.3
Grade			
I	40.9	40.4	8.5
II	30.4	28.9	26.9
III	23.5	26.4	42.4
IV	5.2	4.3	22.2
Familys' economic status			
Rich	14.9	14.5	10.8
Between	73.7	79.1	82.2
Poor	11.4	6.4	7.0
Hometown area			
Countryside	24.6	19.1	27.3
Town/city	75.4	80.9	72.7
Mean age of first sex			
± SD^b^	15.53 ± 1.94	18.38 ± 1.25	20.44 ± 1.16

^a ^Data were shown in percentages, and the perentages of some items may not add up to 100 due to missing data.

^b ^SD, standard deviation.

### Sexual behaviors and risks

At the first experience of intercourse, the proportion of sexually active male students who had sex with a non-regular partner (a casual or a commercial partner), was significantly greater in early than in late sexual initiators. The proportion of non-regular partners in BHS initiators was 35.7%, but it was only 10.2% in Univ initiators. An opposite trend was detected in condom use at first sex: 15.7% of BHS initiators and 32.9% of Univ initiators used condoms (Table 2).

**Table 2:** Timing of sex initiation and its association with subesequent sexual behaviors and risks.

Variables	Total (n = 1908)^a^	BHS (n = 115)^a^	HS (n = 705)^a^	Univ (n = 1088)^a^	*P *value^b^
**First sex**					
Partner type					
Regular	85.4	60.9	83.4	89.2	
Non-regualr	13.9	35.7	16.2	10.2	< 0.001
Condom use					
Used	29.8	15.7	27.2	32.9	< 0.001
Not used/unsure	69.8	84.3	72.2	66.6	
**Most recent sex**					
Partner type					
Regular	78.6	62.6	78.0	80.7	
Non-regualr	10.5	26.1	11.2	8.4	< 0.001
Condom use					
Used	44.2	32.2	42.3	46.7	0.002
Not use	46.9	59.1	48.9	44.3	
OC use by partner					
Used	23.8	21.7	23.3	24.4	0.429
Not use	66.1	70.4	66.4	65.4	
**Sex during last year**^c^					
Partner type					
Only regular	83.0	55.0	81.8	86.5	
Ever non-regular	14.7	40.0	17.4	10.6	< 0.001
Condom use					
Always/often	40.3	18.8	35.6	45.2	< 0.001
Never/rarely/sometime	55.1	76.3	60.4	49.9	
OC use by partner					
Always/often	22.3	13.8	20.2	24.4	0.003
Never/rarely/sometime	74.8	83.8	77.8	72.1	
Partner number					
1	74.1	33.8	66.0	82.9	
≥ 2	17.6	40.0	25.6	10.6	< 0.001
**Sex over lifetime**					
Condom use					
Always/often	41.7	20.9	38.3	46.1	< 0.001
Never/rarely/sometime	52.8	74.8	55.9	48.4	
Partner number					
1	56.9	15.7	40.7	71.8	
≥ 2	32.1	67.0	47.9	18.1	< 0.001
Anal sex					
Yes	3.9	12.2	4.3	2.8	< 0.001
No	90.7	83.5	90.1	91.8	
Partner's pregancy					
Yes	10.2	24.3	10.2	8.6	< 0.001
No/unsure	85.3	70.4	84.8	87.2	
Partner's induced abortion					
Yes	9.6	23.5	9.8	8.0	< 0.001
No/unsure	85.6	70.4	84.7	87.8	
Diagnosed with an STD					
Yes	1.3	7.0	1.6	0.6	< 0.001
No	89.3	86.1	87.8	90.5	

^a ^Data were shown in percentages, and the perentages of some items may not add up to 100 due to missing data.

^b ^Chi square test for linear trend in proportion.

^c ^n = 1409, 80, 500, 829, for Total, BHS, HS, Univ initiator, respectively.

In their most recent sexual experience, the proportion having sex with a non-regular partner was 26.1% in BHS initiators and 8.4% in Univ initiators, whereas the proportion of condom use in their most recent sexual activity was 32.2% in BHS initiators and 46.7% in Univ initiators. Oral contraceptive (OC) use by female partners was slightly higher in BHS initiators than in Univ initiators, although no significant difference was detected between them.

Among sexually active males, 80 of BHS, 500 of HS, and 829 of Univ initiators were sexually active in the last year (69.6%, 70.9%, and 76.1%, respectively). In the last year, early initiators were significantly more likely to have ever had non-regular partners than late initiators; the proportion ever having had a non-regular partner for BHS initiators was nearly four times that of Univ initiators (40.0% vs. 10.6%). Multiple partners were more prevalent among early than late initiators; the proportion was 40.0% in BHS initiators and10.6% in Univ initiators. Early initiators also reported being less likely to have often/always used condoms in the last year; the proportion was 18.8% in BHS initiators and 45.2% in Univ initiators. Early initiators also reported a lower likelihood that their female partner used OCs than did late initiators; the proportion of a partner's always/often using OC in BHS initiators was 13.8%, whereas it was 24.4% in Univ initiators.

With regard to sex during their lifetime, similar to sexual behaviors in the last year, early initiators were significantly more likely to have had multiple partners over their lifetimes and to have used condoms less frequently than late initiators. Anal sex was much more commonly conducted by early than late initiators; the rate was 12.2% for BHS initiators, and 2.8% for Univ initiators. Of the participants, 10.2% reported that they had impregnated a female partner. The prevalence was 24.3% for BHS initiators and 8.6% for Univ initiators; this trend and proportion were similar to female partners' reports of induced abortion. Although the reported diagnosed STD prevalence was generally low, BHS initiators were over ten times more likely than Univ initiators to report having been diagnosed with an STD (7.0% vs. 0.6%).

A multivariate logistic regression model examining the relationship between early sexual initiation and sexual risk and controlling for possible confounding by university, grade, faculty, hometown area, and perceived family economic status, confirmed all trends from the bivariate analyses, showing that early sexual initiation was more likely to be associated with risky sexual behaviors and subsequent consequences. With regard to lifetime sexual behavior and that during the last year, after further adjusting for duration of sexual experience, all the trends showing an increased risk for early sexual initiation compared to late initiation remained, with the majority having an odds ratio > 2, except condom use during the most recent sex activity and number of partners in the last year (an odds ratio of around 1.5); with respect to partner type during the last year, introduction of this adjustment resulted in no such trend (Table 3).

**Table 3:** Multivariate analyses assessing the effects of timing of sexual initiation on subsequent sexual behavior and risks.

Variables	BHS	HS	Univ
	
	Adjusted Odds Ratio (95% Confidence Interval)		
Partner type first sex^a^			
Non-regular vs. regular	5.24 (3.28–8.36)	1.90 (1.40–2.59)	1.00
Condom use first sex^a^			
Not used/unsure vs. used	2.55 (1.50–4.33)	1.30 (1.03–1.64)	1.00
Partner type recent sex^a^			
Non-regular vs. regular	3.70(2.22–6.15)	1.36 (0.96–1.95)	1.00
Condom use recent sex^a^			
Not used vs. used	1.62 (1.05–2.52)	1.06 (0.85–1.33)	1.00
Partner type last year^b^			
Non-regular vs. regular	1.01 (0.40–2.54)	0.96 (0.61–1.53)	1.00
Condom use last year^b^			
Never/rarely/sometime vs. always/often	3.28 (1.36–7.89)	1.24 (0.88–1.73)	1.00
OC use by partner last year^b^			
Never/rarely/sometime vs. always/often	2.68 (1.03–6.98)	1.40 (0.96–2.06)	1.00
Partner number last year^b^			
≥ 2 vs. 1	1.44 (0.58–3.62)	1.43 (0.93–2.21)	1.00
Condom use lifetime^b^			
Never/rarely/sometimes vs. always/often	3.55 (1.74–7.26)	1.17 (0.88–1.56)	1.00
Partner number lifetime^b^			
≥ 2 vs.1	4.19 (1.84–9.54)	2.81 (2.02–3.89)	1.00
Anal sex lifetime^b^			
Yes vs. no	2.28 (0.63–8.23)	1.15 (0.55–2.42)	1.00
Partner's pregancy lifetime^b^			
Yes vs. no/unsure	2.89 (1.21–6.94)	1.42 (0.91–2.22)	1.00
Partner's induced abortion lifetime^b^			
Yes vs. no/unsure	2.95 (1.20–7.26)	1.48 (0.93–2.36)	1.00
Diagnosed with an STD lifetime^b^			
Yes vs. no	26.13 (2.94–232.09)	5.11 (1.20–2.76)	1.00

^a ^Odds ratio with 95% confidence interval in parentheses is adjusted for university, grade, faculty, hometown area, and family economic status.

^b ^Odds ratio with 95% confidence interval in parentheses is further adjusted for the duration of sexual experience.

## Discussion

The results of this study show that young people who initiated sexual activity early were at greater risk for a wide range of sexual and reproductive health problems. Our data showed a clear trend indicating that early onset of sexual activity was associated with increased STD infection, pregnancy, induced abortion, multiplicity of partners, and reduced condom and OC use. The higher incidence of risky behaviors and reproductive health problems attributable to early sexual initiation over a lifetime and sexual behavior in the most recent year may also be explainable by other factors, such as the length of sexual activity. However, the results of our multivariate analyses and indices of recent sexual behavior establish that this is not the whole picture.

In the study sample, the mean age of first sexual intercourse for BHS and HS initiators was 15.5 and 18.4 years, respectively, indicating that most BHS and HS initiators initiated sexual activity during adolescence; 6% of sexually active students initiated sexual activity before high school, and 37% did so in high school. These data show that it is important to conduct effective safe sex education for Chinese students at an early age. Thus, current condom education in China, typically conducted at universities [15], may simply be too late.

We also found that the prevalence of non-regular sex (i.e., sex with casual or commercial partners) at the time of the first experience of intercourse increased dramatically from late to early initiators; this trend was also consistent in the most recent year's behavior and the most recent sexual activity. We found that males whose first sexual experience was non-regular were then more likely to engage in non-regular sex and more likely to have multiple sexual partners during later sexual activity (data not shown). This may indicate that early initiators continued with their partner patterns after their first sexual experience. We also found that young people who began sexual activity in an early school period had an increased likelihood of having multiple partners during the past year and over their lifetime, relative to late initiators, which is consistent with results in other countries [16-20]. This effect persisted even after adjustment for the duration of sexual experience, suggesting that the multiple partnerships of early initiators were not due solely to the longer duration of their sexual experience. Indeed, these early sexual initiators may be more inclined to have multiple partners in their later sexual life than are late initiators. The data highlight that delay in the age of first sexual activity is an important element in reducing non-regular sex and the number of sexual partners; this is important because, among the early initiators, few precautions were taken despite risky sexual behaviors. Our data suggest that early initiators are less likely to adopt responsible behaviors than late initiators, similar to findings in other countries [16,21,22]. The trend for early initiators to use condoms less in sexual activity was seen regardless of time period; these participants seemed not to be worried about STDs/HIV infection in themselves or their partners, or about pregnancy. The quite low rate of condom use in this group increased their risk of acquiring STDs and HIV. Condoms are widely available and affordable in China due to national family planning programs. Why these young initiators had such low condom use is unclear. It may be that early initiators, because of their youth, are less aware of the risks, embarrassed to obtain condoms, or lack the confidence or skill to negotiate condom use with their partners. Furthermore, they may not be prepared when they first engage in sexual activity; thus they do not use condoms then, and once non-protection is established, they continue in this manner, as has been reported in other countries [23,24].

Our results reveal that the earlier male students had initiated sexual activity, the more likely it was that their female partner had experienced pregnancy and induced abortion; similar findings have been reported in other countries, namely that early initiation of sexual activity was associated with pregnancy [16,20,25]. Because this trend remained even after controlling for the length of sexual activity, and because it was also consistent with the lower use of condoms and OC among early initiators compared with late initiators, the higher rates of pregnancy and induced abortions are not explained simply by longer exposure to sexual activity among the early initiators; indeed, the reduced use of oral contraceptives and condoms appears to be the key reason.

Although the reported diagnosed STD rate was low, early sexual activity was associated with increased prevalence of STDs, compared with late initiators. This higher rate of STDs is consistent with lower condom use among early initiators, suggesting that age at first intercourse is a marker for a history of STDs, as has been reported in research in other countries [26,27]. STDs can enhance the transmission of HIV; the early initiators who contracted STDs in this study were particularly vulnerable to HIV infection due to their own risky behaviors and their STD status.

We found a complex risky sexual profile that increased the risks of STDs/HIV infection in early initiators once they started sexual activity. Given the continuing expansion of the HIV/STD epidemic and the rapid social and economic changes in China, if more people initiate sexual activity at an earlier age, the spread of HIV and STDs can be expected to accelerate. Additionally, these male early initiators put both their female partners and themselves at greater risk of HIV/STD infection. Evidence from other countries has shown that HIV transmission from males to females is two to three times more common than from females to males [28-31].

This study has several limitations. Our sample was from a cross-sectional study; whether early sexual initiation is a cause of a male's future risk or whether early sexual initiation increases the prevalence of other identified risky behaviors and reproductive health problems could not be determined from this study. Prospective studies are needed to address this question. Measurements of sexual activity in this study were based on self-reports; participant sensitivity regarding sexual behavior may have led to reporting bias. Additionally, misreporting of sexual behaviors is a recognized problem in sexual-behavior surveys [32]. Indeed, this was evident for some items in this study, with missing data being as high as 11%, which may have affected the power of testing and biased our results; however, we do not believe this lessens the validity of the overall conclusions of the study. Finally, the length of sexual experience was calculated by subtracting the age at first sexual activity from the current age. Because our sample consisted entirely of young people within a narrow age range, the value of using this adjustment may be limited. Additionally, any students who reported their current age and age of first sexual activity as being in the same year would have had a sexual lifetime of zero, which was clearly not the case; this applied to 26.6% of the 1908 sexually active students, which may have led to some bias in the results of the multivariate analysis.

## Conclusion

Our results show that early sexual initiation was a significant predictor of unwanted pregnancy, induced abortion, and STD/HIV infection, emphasizing that controlling the age of first sex is important to reduce these risks. Since education is compulsory until high school in China, to ensure that sex-education programs reach all adolescents in time to encourage delaying sexual activity, junior high school-based programs may be an effective avenue for reaching this target group before they start engaging in sexual activity. Given the rapid change in sexual attitudes and behavior that is occurring among Chinese youth, it should be kept in mind that sex education at different stages of schooling is important in interrupting the transmission of STD/HIV and reducing unwanted pregnancy in this population. Furthermore, such education should address not only delaying first sexual activity but also other issues such as promoting the use of condoms, reducing the number of sexual partners, and addressing other factors that predispose young people to engage in risky sexual behaviors.

## Competing interests

The authors declare that they have no competing interests.

## Authors' contributions

QM performed the statistical analysis and drafted the manuscript; LC and GX coordinated the study in field; QM, PX, and DZ played a major role in the field survey; SZ, SMR and TH helped analyze the data; MOK and MK supervised the research, statistical analysis and revised the manuscript. All the authors read and approved the contents of the manuscript.

## Pre-publication history

The pre-publication history for this paper can be accessed here:


